# Care-seeking behavior and out-of-pocket expenditure for sick newborns among urban poor in Lucknow, northern India: a prospective follow-up study

**DOI:** 10.1186/1472-6963-9-61

**Published:** 2009-04-02

**Authors:** Neeraj M Srivastava, Shally Awasthi, Girdhar G Agarwal

**Affiliations:** 1 Department of Pediatrics, Chattrapati Shahuji Maharaj Medical University, Lucknow 226 003, Uttar Pradesh, India; 2 Department of Statistics, University of Lucknow, Lucknow, Uttar Pradesh, India

## Abstract

**Background:**

The state of Uttar Pradesh, India accounts for one-quarter of India's neonatal deaths and 8 percent of those worldwide. More than half (52%) of these deaths occur due to infections. In order to achieve Millennium Development Goal-4 of reducing child mortality by two-thirds by the year 2015, it is important to study factors which affect neonatal health. In Uttar Pradesh there is meager data for spending on health care in general and neonates in particular.

**Methods:**

The study was conducted at an urban Reproductive and Child Health (RCH) center and a District hospital. Neonates were enrolled within 48 hours of birth and were followed-up once at 6 weeks ± 15 days at the OPD of the respective hospitals or at home. This study assessed (1) distribution of neonatal illnesses and different health providers sought (2) distribution of out-of-pocket expenditures by type of illness and type of health provider sought (3) socio-economic distribution of neonatal illnesses, care-seeking behavior and out-of-pocket expenditures. Per-protocol analysis was performed.

**Results:**

Five hundred and ten neonates were enrolled and 481(94.4%) were followed-up. Parents of 50.3% (242/481) neonates reported at least one symptom of illness. Of these 22.3% (107/481) neonates had illnesses with at least one reported Integrated Management of Neonatal and Childhood Illnesses (IMNCI) danger sign. Among IMNCI illnesses, point prevalence of septicemia was 6.2% and pneumonia was 5.2% while among non-IMNCI illnesses point prevalence of upper respiratory infection was 9.5%, and diarrhea was 7%. Community based non-government dispensers (NGDs) were leading health providers (37.6%). Mean monthly income of families was 2804 Indian Rupees (INR) (range: 800 to 14000; n = 510), where US$ 1 = 42 INR. Mean out-of-pocket expenditure on neonatal illness was 547.5 INR (range: 1 to 15000; n = 202) and mean out-of-pocket expenditure for hospitalization was 4993 INR (range: 41 to 15000; n = 17). All hospitalizations were for IMNCI illnesses. Neonates from lower income strata were less likely to receive any medical care (p < 0.0001) and were also less likely to be seen by a Government provider (p = 0.03).

**Conclusion:**

Since more than half of the neonates have morbidity and out-of-pocket expenditure on neonatal illnesses often exceeds the family income of the lower strata of the low income group in the community, there is a need to either introduce health insurance scheme or subsidize health care for them. Also, since NGDs, half of which could be unqualified are leading health providers, qualified medical care-seeking for sick newborns should be promoted in urban Lucknow.

## Background

Every year, 1.2 million neonates die in India which accounts for more than one-quarter of all neonatal deaths in the World [1]. In India, neonatal deaths constitute two-third of infant deaths and more than half (52%) of these occur due to infections [1].

In order to achieve Millennium Development Goal-4 of reducing child mortality by two-thirds by the year 2015, it is important to study distribution of neonatal illnesses, care-seeking behavior, and direct enabling and disabling factors related to health systems which affect neonatal health [2].

Although, there are few works which have dealt with different behavioral aspects related to care-seeking for sick newborns [3-6], quantitative information is lacking on the inequalities that exist in developing countries in terms of health status or health care utilization [7] especially for newborns [8]. While, there are some works from developing countries which have measured out-of-pocket expenditure incurred by families in response to childhood illnesses [9,10], information is lacking on out-of-pocket expenditure for neonatal illnesses, factors determining it and its effect on neonatal health.

This study is a part of a trial, being performed to assess the effect of behavior change communication on care-seeking behavior for sick neonates in urban Lucknow, northern India.

The present study assesses (1) distribution of neonatal illnesses and different health providers sought. (2) distribution of out-of-pocket expenditures by type of illness and type of health provider sought (3) socio-economic distribution of neonatal illnesses, care-seeking behavior and out-of-pocket expenditures.

## Methods

### Study design

This was a prospective follow-up study of neonates delivered at two public hospitals in urban Lucknow.

### Setting

Lucknow is the capital city of Uttar Pradesh, a state in Northern India and has a native population of 2.2 million [11]. United Nations Centre for Human Settlements (UNCHS) estimates that currently more than half of Lucknow's urban population lives in slums [11]. Nearly 80% of the households in the urban slums of Lucknow have monthly incomes of less than 2000 Indian Rupees (INR) (1 US$ = 42 INR) and approximately 30% of this is spent on health care [11]. Literacy rate in Lucknow is 67.46% and sex ratio is 893 females per thousand males. Institutional delivery rate (59.5%) and antenatal care coverage (54.9%) are much higher than the average in Uttar Pradesh [12]. However, neonatal mortality rate in Lucknow is 51 per thousand and is close to the average of 53.6 in Uttar Pradesh [13].

The study was conducted in two public hospitals, an urban Reproductive and Child Health (RCH) center and a district hospital, after obtaining institutional ethical clearance and permission from relevant district authorities.

The RCH center is a 12 bedded hospital, with free outpatients' clinic and free normal vaginal delivery care facilities round the clock which caters to mainly slum and low income group population from the adjoining areas. District hospital is a large 150 bedded hospital, which is well equipped in dealing with complicated obstetric cases referred from Lucknow and adjoining areas. While the district hospital has paid as well as free inpatient facilities, for this study we have recruited mothers from the free facility only, which caters to mainly lower income group so that economic status of subjects were similar to those recruited from the RCH center.

### Participants and definitions

Neonates were screened within 48 hours of delivery on all working days excluding Sundays and holidays. Mothers, most (97.3%) of which were willing to come to the outpatients' department (OPD) with the baby for follow-up on pre-specified dates in the next 6–8 weeks for DPT immunization were enrolled, after obtaining written informed consent. Excluded were the mothers whose neonates required any resuscitation at birth or presented with any clinically detectable serious congenital malformation at birth. Also, mothers who were not residents of Lucknow or who were likely to move out of the city in next one month were also excluded.

Mothers were requested to keep prescriptions, receipts, wrappers, investigation reports, bottles of medicines etc in case their newborn suffered any illness and were informed that this information would be collected at follow-up.

At the time of follow-up mothers/caregivers were asked if their baby faced any health problem in the neonatal period and if answered in affirmation, symptoms of morbidity were recorded as narrated by them. A structured and pre-tested questionnaire was used to elaborate the details of illnesses as told by the mother. We used simple clinical definitions of neonatal illnesses applicable in field settings, from those recommended by the Integrated Management of Neonatal and Childhood Illnesses (IMNCI) [14] and National Neonatology Forum of India [15]. IMNCI definitions were used for classifying patients as having diarrhea with dehydration, persistent diarrhea, pneumonia, meningitis, ear discharge, fever, more than ten pustules, jaundice and sepsis [16]. IMNCI danger signs were the signs presented in a neonate likely to be suffering from serious bacterial infection, severe jaundice, diarrhea with dehydration or severe persistent diarrhea. Non-IMNCI illnesses were defined and classified as per the guidelines of National Neonatology Forum of India [14]. Non-IMNCI illnesses were upper respiratory tract infection (URI), diarrhea, conjunctivitis, dermatitis, physiological jaundice and others. A comprehensive list of illnesses and symptoms was prepared in local terminology to avoid any translation problem.

Mothers were also enquired about the health care provider/providers consulted, expenditure incurred on consultation, medicine, investigations, hospitalization (if any) and conveyance. Medical provider's prescriptions, investigation records, medicine bottles, chemist's receipts etc, one or more of which were available in all cases, were also taken into account to support the information provided by the caregivers and validate their claims regarding expenditure. If the mother along with the baby did not turn up in the out-patients' department, home follow-up was done by a trained medical social worker to obtain the same information.

We have classified health care providers in 3 categories [9]:

#### (i) Government Physicians (GPs)

Those employed by the government and working through government hospitals. To obtain their services, mothers have to pay only the hospital registration fees. Often medicines were provided free of charge. All the GPs are qualified medical practitioners.

#### (ii) Non Governmental Consultants (NGCs)

These health care providers work through privately owned clinics/hospitals and give formal prescription notes to their patients. Almost all of them have recognized allopathic medical qualification. They charge for consultation and investigations, and prescribed medicines are purchased from a pharmacy.

#### (iii) Non-governmental dispensers (NGDs)

These are also self-employed health care providers. The basic difference between these and the NGC is that the former dispense medicines without prescription. Therefore, there is no record of medicines taken by their patients. Mostly, their service charge is inclusive of the cost of medicine. Unlike the NGCs, the NGDs are a heterogeneous group, some with a degree in allopathic medicine, others with qualifications in indigenous systems of medicine like Ayurveda, Unani and Homeopathy, who may also prescribe allopathic drugs. Yet there are many others who have no recognized qualifications.

### Construction of income strata

We have constructed five strata on the basis of different income groups. These are S1: ≤ 1700 INR (n = 105); S2: 1701–2000 INR (n = 136); S3: 2001–2500 INR (n = 82); S4: 2501–4000 INR (n = 118); S5: > 4000 INR (n = 69). The basis for deciding the cut-off points for these strata were quintile values. However, the strata are exhibiting unequal distribution because several persons were having a particular income equal to the cut-off points, namely quintile values.

### Statistical Analysis

To assess the distribution of different categorical variables among five income strata (S1-S5) in Additional file 1, chi-square test for equality of proportions (H_0_: p_1 _= p_2 _= p_3 _= p_4 _= p_5_) is used. The point prevalence of neonatal illnesses along with 95% confidence intervals have been given in Table 1. The mean, standard deviation along with range have been given for out-of-pocket expenditure for non-hospitalized (Table 2) as well as for hospitalized neonates. To compare the care-seeking expenditure among the three health care-providers (Table 3), Kruskall-Wallis test is used, whereas for pair-wise comparison Mann-Whitney U test is used. Mann Whitney U test is uniformly used wherever pair-wise comparison of incomes or expenditures is done and chi-square test has been used wherever proportions have been compared. We also report crude Odds Ratios (OR) along with 95% confidence intervals and p-values for baseline variables associated with care-seeking behavior. Per-protocol analysis has been performed. For all statistical tests p-value of < 0.05 is taken to be significant.

**Table 1:** Distribution of neonatal illnesses and care-seeking pattern in urban Lucknow (N = 481)

**Neonatal Illnesses**	**Point prevalence** **n (%)(95% CI)**	**Any outside medical care (n)**	**Health Care provider sought**		
			
			**GPs (n)**	**NGCs (n)**	**NGDs (n)**
**IMNCI (n = 107 (22.2%))**					
Diarrhea with dehydration	4 (0.8) (0.3, 2.3)	4	1	3	1
Persistent Diarrhea	7 (1.4) (0.6, 3.1)	6	1	2	3
Ear discharge	4 (0.8) (0.3, 2.3)	4	2	1	1
Fever	14 (2.9) (1.7, 4.9)	4	1	0	3
Pathological jaundice	5 (1.0) (0.4, 2.5)	5	4	1	0
Meningitis	2 (0.4) (0.07, 1.6)	2	2	0	1
Pneumonia	25 (5.2) (3.5, 7.7)	25	5	5	19
Septicemia	30 (6.2) (4.3, 8.8)	30	21	6	10
More than 10 Pustules	11 (2.3) (1.2, 4.2)	7	5	1	2
Umbilical sepsis	5 (1.0) (0.4, 2.5)	4	1	1	2
**Non-IMNCI (n = 135 (28.1%))**					
Conjunctivitis	8 (1.7) (0.8, 3.4)	7	3	3	2
Dermatitis	11 (2.3) (1.2, 4.2)	8	3	1	4
Diarrhea	34 (7.0) (5.0, 9.8)	29	12	6	14
Upper Respiratory Infection	46 (9.5) (7.2, 12.6)	35	6	6	24
Physiological Jaundice	16 (3.3) (1.9, 5.4)	16	11	3	2
Others*	20 (4.1) (2.6, 6.4)	16	8	7	3

**Total**	242 (50.3) (45.7, 54.9)	202	86	46	91

GPs = Government Practitioners; NGCs = Non Government Consultants; NGDs = Non-Government Dispensers

* includes vomiting = 9, oral thrush = 4, evening colic = 2, umbilical granuloma = 2, injury = 2, brachial palsy = 1

**Table 2:** Mean, standard deviation and range of out-of-pocket expenditure^# ^for non-hospitalized neonates having IMNCI or non-IMNCI illnesses (N = 185)

**Neonatal Illnesses**	**Consultation** **Mean (SD) ** **[range]**	**Medicines** **Mean (SD) ** **[range]**	**Conveyance** **Mean (SD) ** **[range]**	**Combined** **Mean (SD)** ** [range]**
**IMNCI**				
Diarrhea with dehydration (n = 3)	61.7 (50.6) [30, 120]	100.0 (50.0) [50, 150]	20.0 (26.5) [0, 50]	181.7 (52.5) [130, 235]
Persistent Diarrhea (n = 6)	65.3 (48.3) [2, 120]	78.3 (84.9) [0, 200]	50.0 (27.5) [20, 100]	193.7 (83.2) [150, 350]
Ear discharge (n = 4)	15.8 (16.9) [1, 35]	11.3 (13.1) [0, 25]	15.0 (10.0) [0, 20]	42.0 (36.1) [1, 75]
Unexplained Fever (n = 4)	42.8 (43.1) [1, 100]	60.0 (48.9) [0, 100]	13.8 (4.7) [10, 20]	116.5 (96.9) [21, 210]
Pneumonia (n = 24)	47.6 (51.2) [1, 200]	86.4 (66.8) [0, 200]	27.4 (27.0) [0, 100]	161.4 (120.6) [30, 370]
Septicemia (n = 22)	34.2 (57.3) [1, 200]	93.8 (115.8) [0, 400]	25.7 (19.4) [0, 50]	153.6 (163.0) [11, 600]
More than ten Pustules (n = 7)	10.9 (12.5) [1, 31]	17.6 (21.7) [0, 58]	20.0 (18.3) [0, 50]	48.4 (35.9) [1, 100]
Umbilical sepsis (n = 4)	15.5 (23.4) [1, 50]	73.7 (87.1) [0, 200]	15.0 (5.8) [10, 20]	104.3 (113.7) [11, 270]
**Non-IMNCI**				
Conjunctivitis (n = 7)	32.3 (46.7) [1, 100]	78.6 (89.8) [0, 250]	24.6 (16.9) [0,50]	135.4 (131.7) [3, 310]
Dermatitis (n = 8)	27.5 (50.3) [1, 150]	61.4 (99.8) [0, 300]	23.8 (23.7) [0, 80]	112.6 (171.2) [21, 530]
Diarrhea (n = 29)	27.2 (30.6) [1, 100]	51.9 (49.4) [0, 156]	20.6 (13.2) [0, 50]	99.7 (75.4) [11, 306]
Upper Respiratory Infection (n = 35)	23.5 (22.5) [1, 100]	41.8 (46.6) [0, 200]	14.5 (14.1) [0, 50]	79.7 (69.4) [1, 350]
Jaundice (n = 16)	18.0 (34.4) [1, 130]	182.8 (69.5) [0,300]	23.8 (12.5) [0,30]	224.6 (123.4) [21,460]
Others (n = 16)	33.4 (49.5) [1, 202]	70.3 (105.6) [0, 400]	19.9 (11.2) [0,40]	123.6 (154.5) [16, 632]

^#^The figures are in Indian Rupees (INR) where 1 US $ = 42 INR

**Table 3:** Comparative expenditure^@ ^incurred in seeking care from different health care providers (N = 168)

**Expenditure**	**GPs (n = 60)** **Mean (SD)** **[range]**	**NGCs (n = 30)** **Mean (SD)** **[range]**	**NGDs (n = 78)** **Mean (SD)** **[range]**	**p value^#^** **(All Groups)**	P value*		
					
					**GPs Vs NGCs**	**GPs Vs NGDs**	**NGCs Vs NGDs**
**Consultation + medicine**	39.3 (34.0) [1, 141]	170.2 (107.3) [30, 450]	89.9 (134.7) [3, 1050]	< 0.001	< 0.001	0.006	< 0.001
**Conveyance**	21.7 (12.8) [0, 50]	23.7(18.4) [0, 80]	18.4 (21.3) [0, 100]	0.03	0.8	0.01	0.06

**Combined**	61.0 (38.2) [1, 161]	193.9 (121.6) [40, 530]	108.3 (145.1) [3, 1150]	< 0.001	< 0.001	0.07	< 0.001

^@ ^the figures are in Indian Rupees (INR) where 1 US$ = 42 INR;^# ^Kruskall Wallis test; * Mann-Whitney U test

## Results

Five Hundred and ten neonates were enrolled (154 from RCH center and 356 from the district hospital) from March 2007 to September 2007 and 481 (94.4%) were followed-up at the OPD (30.2%) of the respective hospitals or at home (64.2%). In all 29 (5.6%) neonates were lost to follow up i.e. those who neither came to OPD nor could be located on field visit.

Among those enrolled, 52.7% (268/510) were males and 36.4% (185/510) were of the first birth order. More than half (52.9%, 269/510) were Hindus and 46.9% (238/510) were Muslims. Mean age of mothers was 24.8 (± 3.8) years and mean age of fathers was 29.2 (± 5.0) years. Mean monthly family income was 2804.5 (± 1565.05) INR. (1 US$ = 42 INR).

### Baseline and outcome variables (Site-wise)

Additional file 1 includes the distribution of baseline as well as outcome (follow-up) variables for the neonates delivered at the RCH center and at the district hospital.

Proportion of slum dwellers was higher among the mothers who delivered at RCH center as compared to those who delivered at the district hospital (p < 0.0001). Mothers as well as fathers from the RCH center were less likely to be educated (p < 0.0001 for both) and more likely to have nuclear families (p = 0.01). Fathers at the RCH center were less likely to work on monthly wages (p = 0.04) and were more likely to be self-employed (p = 0.02) as compared to fathers from the district hospital. Mothers who delivered at the RCH center were less likely (p = 0.004) to have made three antenatal care (ANC) visits and were also less likely to have received ≥ 2 doses of Tetanus Toxoid (p = 0.009). Parity ≥ 4 was more likely (p = 0.01) among mothers who delivered at the RCH center (p = 0.01). Monthly family incomes of those enrolled from RCH center were significantly lower than those enrolled from the district hospital (p < 0.00001). Mean monthly family income was 2337.6 (± 1039.3) INR) among those enrolled from RCH center and 2988.3 (± 1720.3) INR among those enrolled from the district hospital.

Neonatal morbidity was similar across the sites (54.5% *vs *48.5%; p = 0.22). Prevalence of IMNCI illnesses (21.7% (31/143) *vs *22.5% (76/338); p = 0.8) and non-IMNCI illnesses (32.9% (47/143) *vs *26.1% (88/338); p = 0.12) were also similar. Mothers enrolled from the RCH center were less likely to seek any medical care for neonatal illnesses (71.8% *vs *89.1%; p = 0.0007) and were also less likely to seek care from GPs (19.2% *vs *43.3%; p = 0.0002). However, care-seeking from NGDs was similar (42.3% *vs *35.3%; p = 0.29). For cases in which any medical care was sought (excluding hospitalization), families from the RCH center spent less than the families of the district hospital (p = 0.07).

### Baseline and outcome variables (according to income strata)

Additional file 1 also includes distribution of baseline as well as outcome variables across five income strata. Mothers from the lower income strata were more likely to be slum dwellers (p = 0.0005) and were less likely to be educated (p < 0.0001). Fathers from lower income strata were more likely to work on daily wages (p = 0.0002). Families from lower income strata were more likely to be nuclear (p = 0.03). Mothers from lower income strata were less likely to have made ≥ 3 antenatal care (ANC) visits (p = 0.004) and were more likely to have parity ≥ 4 (p = 0.03).

Neonatal morbidity across the five income strata did not differ significantly (p = 0.06). However, mothers from the lower income strata were less likely to seek any medical care for neonatal illnesses (p < 0.0001) and were also less likely to seek care from GPs (p = 0.03).

### Neonatal morbidity and care-seeking behavior (overall)

Table 1 shows the overall distribution of neonatal illnesses and health providers sought. More than half (50.3% (242/481)) developed any illness in the neonatal period. Of these 22.3% (107/481) developed an illness which incorporated one or more IMNCI danger signs while 28.1% (135/481) had an illness associated with non-IMNCI signs. Mean number of illnesses per neonate was 1.09 (± 0.28), n = 242. We have considered only primary illnesses for analysis i.e. illnesses more likely to cause neonatal mortality (such as IMNCI illnesses) as per the available literature have been selected in cases of multiple illnesses (n = 13). Cases where two non-IMNCI illnesses were reported, illnesses likely to incur higher medical expenditure were selected (n = 9).

More than four-fifths (83.5% (202/242)) of the sick neonates received any type of medical care, which was similar for IMNCI (91/107, 85.0%) and non-IMNCI illnesses (111/135, 82.2%), respectively.

Overall, 35.5% (86/242), 19.0% (46/242) and 37.6% (91/242) neonates were shown to GPs, NGCs and NGDs respectively. We observed that people preferred NGDs for pneumonia (76% (19/25)) and URI (68.6% (24/35)) while GPs were preferred for septicemia (70% (21/30)) and any type of jaundice (80% (4/5) for pathological jaundice and 68.8% (11/16) for physiological jaundice, respectively).

More than one health care provider was approached for 10.4% (21/202) neonates and hence they have appeared more than once under different care seeking types in the Table 1. There were 17 hospitalizations, all for IMNCI illnesses. Fourteen out of seventeen hospitalizations were done at government hospitals. Many medicines and procedures in these cases were provided free of cost. All cases of pathological jaundice (5/5), meningitis (2/2) and 26.6% (8/30) cases of septicemia (including 2 cases of necrotizing enterocolitis) were hospitalized along with one case each for diarrhea with dehydration and pneumonia.

### Neonatal deaths

There were 6 neonatal deaths and 2 infant deaths (4–6 weeks) in our study. Among these, no care was sought for one neonate due to perceived rapid course of disease progression by the caregivers. Of the remaining five, one expired in a government and the other in a private hospital (both from necrotizing enterocolitis), the latter was being seen by a NGD before being referred to a hospital. Three neonates (probable septicemia) were being treated by NGDs on a domiciliary basis, who failed to refer the babies to tertiary care. Among two infant deaths, no care was sought for one infant who died of pneumonia while the other infant who died of probable septicemia was being treated by a NGD on domiciliary basis.

### Expenditure on Illness

#### For non-hospitalized neonates (n = 185)

Out-of-pocket expenditures for IMNCI illnesses which were diarrhea with dehydration, persistent diarrhea, ear discharge, fever, pneumonia, septicemia, more than 10 pustules and umbilical sepsis have been shown in Table 2, along with out-of-pocket expenditures on non-IMNCI illnesses which were conjunctivitis, dermatitis, diarrhea, upper respiratory tract infection, jaundice and others. Combined mean expenditure on non-hospitalized neonates with IMNCI illnesses was 159.9 INR (range 1 to 1150; n = 74) and did not differ significantly from expenditure incurred on non-IMNCI illnesses (121.6 INR (range: 1 to 832; n = 111), (p = 0.08). The costs of consultation could be a little lower and cost of medicines could be a little higher than depicted in Table 2 as cost of consultations included cost of medicines for many cases in which care was sought from NGDs. Also cost of medicines in Table 2 includes costs of investigations done, if any. Among non-hospitalized neonates, 2 were investigated for septicemia, 1 for pneumonia, 13 for physiological jaundice and 1 for injury.

#### For hospitalized neonates (n = 17)

Seventeen (3.5%, 17/481) neonates were hospitalized for septicemia (n = 6), pathological jaundice (n = 5), meningitis (n = 2), necrotizing enterocolitis (n = 2), pneumonia (n = 1) and diarrhea with dehydration (n = 1). Here, mean combined expenditure for septicemia was 5048 INR (SD: 5255; range 390 to 14180), for pathological jaundice was 2968 INR (SD: 2675; range 41 to 5410), for meningitis was 9750 INR (range 4501 to 15000), for necrotizing enterocolitis was 8250 INR (range 5000 to 11501), for pneumonia was 1151 INR and for diarrhea with dehydration was 2600 INR. Overall mean combined expenditure on hospitalized neonates was 4993 INR (SD: 4601; range 41 to 15000, n = 17).

#### Overall expenditure (IMNCI and non-IMNCI) (n = 202)

Combined mean out-of-pocket expenditure on all neonatal illnesses was 545.7 INR (range: 1 to 15000, n = 202). Combined mean out-of-pocket expenditure on all IMNCI illnesses was 903.9 INR (range: 1 to 15000; n = 91) while for all non-IMNCI illnesses was 121.6 INR (range: 1 to 832; n = 111) (p < 0.001).

IMNCI illnesses were significantly more among males ((67/249) (26.9%) Vs (40/232) (17.2%); p = 0.01) but out-of-pocket expenditures for males (mean = 645.36 INR; range: 1 to 15000; n = 122) and for female neonates (mean = 380.83 INR; range = 3 to 6501; n = 80) did not differ statistically (p = 0.5). In total, 10 males and 7 females were hospitalized. Among our study group 4 male neonates and 2 female neonates died.

Among those who fell ill, 9.4% (7/74) of the sick low birth weight (LBW) neonates were hospitalized, while 25.9% (7/27) of sick preterm neonates and 28.6% (6/21) of the neonates who were both LBW and preterm were hospitalized. Here, it should be noted that these are overlapping categories.

Mean out-of-pocket expenditure on treatment of sick LBW neonates was 593.1 INR (range: 2 to 11501; n = 60). Mean expenditure on treatment for sick pre-term babies (< 37 weeks) was 1272.2 INR (range: 2 to 11501; n = 33) and for neonates who were both LBW and preterm was 1487.9 INR (range: 2 to 11501, n = 20).

### Association of baseline variables with care-seeking from any health provider

Mothers who made less than 3 antenatal visits (i.e. 0 to 2 visits) were less likely to seek any medical care for their sick newborns as compared to those mothers who had ≥ 3 antenatal visits (OR = 3.2, 95% CI = 1.45 to 7.1, p = 0.001). Also, mothers who had a parity of ≥ 4 were less likely to seek care as compared to mothers with parity less than four (OR = 2.16, 95% CI = 0.87 to 5.3, p = 0.06), although this was not statistically significant.

Neonatal morbidity and care-seeking from any health provider was significantly higher for preterm (< 37 weeks) neonates as compared to term neonates (OR = 3.34, 95% CI = 1.43 to 8.0, p = 0.001) and (OR = undefined; p = 0.008) respectively. However, neonatal morbidity and care-seeking behavior was similar for low birth weight (LBW) and non-LBW babies respectively. Reported neonatal illnesses were also higher among males as compared to females (OR = 2.1, 95% CI = 1.5 to 3.2, p < 0.001) but care-seeking was similar across genders.

### Neonatal morbidity and care-seeking behavior according to Income group

Since the median income in our study group was 2500 INR, we have combined S1, S2 and S3 as group A i.e. strata having income ≤ 2500 INR and compared it with combined higher income strata (S4, S5) (group B) i.e. strata having income more than 2500 INR (Additional file 1).

Neonatal morbidity was higher in group A as compared to group B (OR = 1.48, 95% CI = 1.0 to 2.18, p = 0.03) but group A was less likely to seek any medical care as compared to group B (OR = 5.5, 95% CI = 1.8 to 19.1, p = 0.0005) and was also less likely to be seen by a government provider (OR = 2.3, 95% CI = 1.3 to 4.1, p = 0.003).

### Comparative expenditure incurred in seeking care from different health providers

We excluded 17 cases of hospitalization and 17 other cases of multiple care-seeking and analyzed 83.2% (168/202) of the remaining cases which were seen by a single provider. We excluded cases of hospitalization in this analysis because most of the neonates were hospitalized in government hospitals (14/17); hence costs incurred by families could show a wrong measure against the care-seeking from GPs. We also excluded 17 other cases of multiple care-seeking (where 2–3 medical providers were consulted) so as to avoid counting the costs more than once.

It was found that the expenditures incurred by the families in seeking care differed significantly across the groups (Table 3).

We also found that conveyance cost was zero in 29.5% (23/78) cases in which care was sought from NGDs as compared to 6.6% (4/60) cases in GPs and 13.3% (4/30) cases in NGCs (p = 0.002).

It was also found that people were reasonably satisfied by the services NGDs were providing. In our study group, 88.3% (53/60) of the mothers were satisfied with the medical treatment they received by GPs as compared to 80.0% (24/30) for NGCs and 82.1% (64/78) for NGDs respectively (χ^2 ^= 1.41, p = 0.49).

## Discussion

We found that septicemia (6.2%) followed by pneumonia (5.2%) were most prevalent IMNCI illnesses, while upper respiratory infection (9.5%) and diarrhea (7%) were major non-IMNCI illnesses among neonates in urban Lucknow. Outside-the-home care-seeking was high for both IMNCI as well as non-IMNCI illnesses. People preferred NGDs for treatment of pneumonia (76%) and upper respiratory infection (68.6%) while septicemia was preferably shown to GPs (70%). Hospitalization were mostly (82.4%) done at public hospitals. All the hospitalizations were for IMNCI illnesses. Mean out-of-pocket expenditure on neonatal illness was 547.5 INR (range: 1 to 15000; n = 202) and mean out-of-pocket expenditure for hospitalization was 4993 INR (range: 41 to 15000; n = 17).

Although there were significant disparities in baseline variables across different income strata, difference in neonatal morbidity did not achieve statistical significance (p = 0.06). However, we observed that sick newborns among lower income strata were less likely to receive any medical care (p < 0.0001) and were also less likely to be seen by GPs (p = 0.03). We also observed that the mothers who delivered at the RCH center were less likely to seek any medical care for neonatal illnesses (p = 0.0007) and were also less likely to seek care from GPs (p = 0.0002) as compared to mothers who delivered at the district hospital.

It was found that cost of consultation and medicine were significantly lower when care was sought from GPs despite the fact that not all medicines were available at the hospital and external purchase was written in around two-third (39/60) cases. Mean combined out-of-pocket expenditure in seeking care from NGDs was 1.77 times higher and from NGCs was 3 times higher as compared to GPs (p < 0.001). These findings suggest that the out-of-pocket expenditure not only depended on the illness and its severity, but was largely influenced by the type of provider sought.

We found that all the hospitalizations were for IMNCI illnesses and the combined overall mean expenditure incurred on IMNCI illnesses was nearly nine times higher as compared to that on non-IMNCI illnesses (903 INR Vs 121.6 INR). Hence it is important to train care-givers and health workers in recognition of these illness conditions and timely referral so that the progression of severity and eventual mortality could be averted which in turn would be instrumental in bringing down the neonatal mortality and concurrently reduce the costs incurred by the families once the disease gets severe.

We observed that majority (83.4%) of mothers sought care outside the home for neonatal illnesses and NGDs were the leading health providers (37.6%). Mothers could have found care-seeking from NGDs as logistically more feasible as they were easily accessible (in vicinity) and medicines were dispensed at consultation. However, more than half of the NGDs in Lucknow may not have a recognized medical degree [9] and therefore their efficiency in managing newborns is uncertain. This could be one of the many reasons for high neonatal mortality reported from Lucknow and emphasizes the need for promotion of qualified medical care-seeking such as from GPs and NGCs, here.

Role of private sector in health care has witnessed a continuous rise during past two decades [17]. We observed that 56.6% of the mothers in our study group went to private providers (19% to NGCs and 37.6% to NGDs), which was similar to other studies which report frequent use of private sector even by poorer classes [17-19]. Uncertain availability of drugs/services and extremely formal procedures at public hospitals have been previously reported as some of the reasons for preference of private sector [17,19,20]. However, our findings suggest that steps must be taken to promote care-seeking from GPs, as it was significantly economical.

It was also observed that cases of respiratory infections such as pneumonia and URI were mostly shown to NGDs whereas septicemia and jaundice were mostly shown to GPs. Due to high prevalence of acute respiratory infections in children among urban poor, mothers could have considered these episodes as common and just like other episodes seen by them in other children previously. However, further ethnographic studies are required to explore the reasons for this type of differential care-seeking observed by us.

We found that the reported neonatal illnesses were significantly higher for male neonates but we did not find any statistical difference in out-of-pocket expenditures across genders. However, a study done in rural Uttar Pradesh found a gender bias in favor of male neonates both in terms of perception of illness and out-of-pocket expenditures [18].

Our study was done in public hospitals among mothers from low socio-economic groups and therefore cannot be generalized for all institutional deliveries. Moreover, majority of the mothers from our study group had sought antenatal care at least once; hence our findings cannot be generalized for mothers who never seek care for themselves or their newborns. The burden of neonatal morbidity and mortality could be even higher among such groups and this could possibly contribute to high neonatal mortality rate reported from Lucknow [13]. Also since 82.4% (14/17) of the hospitalizations were done at government hospitals where services were subsidized, the cost of care could be much higher and expenditures may be underestimated by us. It should also be noted that this is not a cost-of-illness study since the costs incurred by the health systems have not been calculated and we only report out-of-pocket expenditure incurred by families in seeking care for neonatal illnesses. We have also not assessed the risk factors for neonatal illnesses in this study, as it has been widely researched in developing countries [21-24] and was not an objective of the current study.

Due to poor coverage of any health insurance program, out-of-pocket expenditure contributes to three-quarter of the total expenditure on health care in India [17,19]. Health expenditure on curative care is 6% of the household income and has been estimated to vary from 3% to 12% among highest and lowest income quintiles respectively [17]. At the same time the medical care expenditure made by a poor household in comparison to its expenditure/earning potential is much higher than that of rich household. Although our study group may be assumed to be uniformly poor (i.e. those opting for free institutional deliveries and mean family income 2804.5(± 1565.05) INR), we found that percentage of income spent on curative care for non-hospitalized neonates was 10.3% and 3.8% among lowest and highest income strata respectively.

In our study, mean household income was 2804.5 INR (range: 800 to 14000) while mean out-of-pocket expenditure on neonatal hospitalization was 4993 INR (range: 41 to 15000). More than three-quarter (13/17) of the families who sought inpatient care for their neonates incurred out-of-pocket expenditures exceeding their monthly family incomes. This type of impact of expenditure on health care drastically affects poor households making them even poorer and is a major cause of debts among poor families [17,19].

NGDs constitute largest share in the overall care-seeking for newborns and as they are not covered under Government's training programs, it is important to identify and train them with respect to diagnosis, early management and referral of sick newborns under the Integrated Management of Neonatal and Childhood Illnesses (IMNCI) program.

## Conclusion

We conclude that since more than half of the neonates have morbidity and out-of-pocket expenditure for neonatal illnesses often exceeds the family income of the lower strata of the low income group in the community, there is a need to introduce health insurance scheme or further subsidize health care. Also, since NGDs, half of which could be unqualified are leading health providers, qualified medical care-seeking for sick newborns should be promoted in urban Lucknow.

## Competing interests

The authors declare that they have no competing interests.

## Authors' contributions

SA conceptualized the study and supervised the data collection, analysis and manuscript writing process. NMS collected the data. GA and NMS analyzed the data. All authors helped in the preparation of manuscript. All authors read and approved the final manuscript.

## Pre-publication history

The pre-publication history for this paper can be accessed here:



## Supplementary Material

Additional file 1 **Table S1**. Baseline and follow-up variables for neonates in urban Lucknow (N = 510) Click here for file
