# Supplementary material for: Care-seeking behavior and out-of-pocket expenditure for sick newborns among urban poor in Lucknow, northern India: a prospective follow-up study
Source: BMC Health Serv Res. 2009 Apr 2;9:61. doi: 10.1186/1472-6963-9-61 (PMC2676263; doi:10.1186/1472-6963-9-61)
Supplement: Additional file 1 — Table S1. Baseline and follow-up variables for neonates in urban Lucknow (N = 510) [file 1472-6963-9-61-S1.doc]

**Table 1: Baseline and follow-up variables for neonates in urban Lucknow (N=510**)

| **Variables**  **Baseline** | **Site-wise and overall distribution** | | |  | **Distribution according to five income strata** | | | | | |
| --- | --- | --- | --- | --- | --- | --- | --- | --- | --- | --- |
|  | **Site 1^#^**  **(n=154)**  **m (%)** | **Site 2^#^**  **(n=356)**  **m (%)** | **Total**  **(n=510)**  **m (%)** |  | **S1^$^**  **(n=105)**  **m (%)** | **S2^$^**  **(n=136)**  **m (%)** | **S3^$^**  **(n=82)**  **m (%)** | **S4^$^**  **(n=118)**  **m (%)** | **S5^$^**  **(n=69)**  **m (%)** | **χ2 , p-value** |
| **Residence (slum)** | 74(48.1) | 90(25.3) | 164(32.1) |  | 49 (46.6) | 47 (34.5) | 26 (31.7) | 30 (25.4) | 12 (17.4) | 19.6, 0.0005 |
| **Education**   1. Mothers (no formal education) 2. Fathers (no formal education) | 74(48.1)  82(53.2) | 87(24.4)  81(22.7) | 161(31.6)  163(32.0) |  | 51 (48.6)  53 (50.5) | 63(46.3)  56(41.2) | 21(25.6)  27(32.9) | 22(18.6)  26(22.1) | 4(5.7)  1(1.4) | 60.4, <0.0001  57.0, <0.0001 |
| **Occupation**   1. Mothers (unemployed) 2. Fathers    1. daily wages    2. monthly wages/professionals    3. self employed    4. Others | 146(94.8)  22(14.3)  28(18.2)  104(67.5)  00(0.0) | 342(96.1)  41(11.5)  95(26.7)  201(56.5)  19(5.3) | 488(95.6)  63(12.4)  123(20.6)  305(59.8)  19(3.7) |  | 100 (94.2)  22 (20.9)  13 (12.3)  59 (56.2)  11 (10.4) | 129 (94.8)  22 (16.2)  35 (25.7)  71 (52.2)  08 (5.9) | 80 (98.7)  11 (13.4)  20 (24.4)  51 (62.2)  00 (00) | 115(97.4)  07 (5.9)  31 (26.3)  80 (67.8)  00 (00) | 64 (92.7)  01 (1.4)  24 (34.8)  44 (63.8)  00 (00) | 3.2, 0.5  21.1, 0.0002  12.6, 0.01  7.6, 0.1  --- |
| **Mother’s age**  < 20 completed years | 21(13.6) | 42(11.7) | 63(12.3) |  | 16 (15.2) | 15 (11.1) | 12 (14.6) | 15 (12.7) | 5 (7.2) | 3.1, 0.5 |
| **Family structure**  (nuclear) | 49(31.8) | 75(21.1) | 124(24.3) |  | 37 (35.2) | 32 (23.5) | 14 (17.1) | 28 (23.7) | 13 (18.8) | 10.3, 0.03 |
| **No. of ANC visits**  (< 3visits i.e. 0-2)  **Tetanus Toxoid**  (<2 doses i.e. 0-1) | 48(31.1)  26(16.9) | 68(19.1)  31(8.7) | 116(22.7)  57(11.2) |  | 32 (30.5)  18 (17.1) | 40 (29.4)  17 (12.5) | 15 (18.3)  7 (8.5) | 22 (18.6)  10 (8.4) | 7 (10.1)  5 (7.2) | 15.3, 0.004  6.5, 0.1 |
| **Parity**  (> 4)  **Prior still births** | 31(20.1)  4(2.6) | 41(11.5)  12(3.4) | 72(14.1)  16(3.1) |  | 19 (18.1)  07 (6.7) | 26 (19.1)  05 (3.7) | 9 (10.9)  02 (2.4) | 15 (12.7)  01 (0.8) | 3 (4.3)  01 (1.4) | 10.5, 0.03  7.3, 0.1 |
| **Birth weight(< 2500 grams)** | 37(24.0) | 111(31.2) | 148(29.0) |  | 36 (34.1) | 43(31.6) | 23(28.1) | 30(25.4) | 16(23.2) | 3.9, 0.4 |
| **Followed-Up** | **(n=143)** | **(n=338)** | **(n=481)** |  | **(n=94)** | **(n=125)** | **(n=79)** | **(n=115)** | **(n=68)** |  |
| **Neonatal morbidity** | 78(54.5) | 164(48.5) | 242(50.3) |  | 52 (55.3) | 63 (50.4) | 46 (58.2) | 57 (49.6) | 24 (35.3) | 9.1, 0.06 |
| **Care-seeking behavior***   1. GPs 2. NGCs 3. NGDs 4. No medical care | 15(19.2)  10(12.8)  33(42.3)  22(28.2) | 71(43.3)  36(21.9)  58(35.3)  18(10.9) | 86(35.5)  46(19.0)  91(37.6)  40(16.5) |  | 18 (34.6)  12 (23.1)  24 (46.2)  06 (11.5) | 15 (23.8)  08 (12.7)  26 (41.3)  18 (28.6) | 14 (30.4)  07 (15.2)  15 (32.6)  12 (26.1) | 29 (50.9)  10 (17.5)  19 (33.3)  03 (5.3) | 10 (41.7)  09 (37.5)  07 (29.2)  01 (4.2) | 10.6, 0.03  8.0, 0.09  3.6, 0.4  18.5, <0.0001 |
| **Hospitalization** | 03(2.1) | 14(4.1) | 17(3.5) |  | 4 (4.3) | 5 (4.0) | 2 (2.5) | 2 (1.7) | 4 (5.7) | 2.5, 0.6 |
| **Newborn mortality** | 02(1.4) | 04(1.2) | 06(1.2) |  | 4 (4.3) | 2 (1.6) | 0 | 0 | 0 | ---- |
| **% of monthly income spent on neonatal illness** (excluding hospitalization) | 5.1 | 5.0 | 5.0 |  | 10.3 | 6.9 | 4.1 | 3.7 | 3.8 | ---- |

^#^Site 1= Reproductive and Child Health (RCH) Center; ^#^Site 2= District hospital. ^$^S1, S2, S3, S4, S5 are lowest to highest income strata respectively. ANC = Antenatal Care; GPs = Government Practitioners; NGCs = Non-government qualified Consultants; NGDs= Non-Government Dispensers; * more than one provider could be consulted for any morbidity; hence sum of numbers in care-seeking behavior columns may be more than the number in the neonatal morbidity columns
